# Effectiveness of Augmented and Virtual Reality-Based Interventions in Improving Knowledge, Attitudes, Empathy and Stigma Regarding People with Mental Illnesses—A Scoping Review

**DOI:** 10.3390/jpm13010112

**Published:** 2023-01-04

**Authors:** Jing Ling Tay, Huiting Xie, Kang Sim

**Affiliations:** 1 Institute of Mental Health, West Region, Buangkok Green Medical Park, Singapore 539747, Singapore; 2 Institute of Mental Health, Buangkok Green Medical Park, Singapore 539747, Singapore; 3 Yong Loo Lin School of Medicine, National University of Singapore, Singapore 117597, Singapore; 4 Lee Kong Chian School of Medicine, Nanyang Technological University, Clinical Sciences, Singapore 308232, Singapore

**Keywords:** virtual reality, augmented reality, mental health literacy, help-seeking, empathy, stigma

## Abstract

Interventions adopting augmented and virtual reality (AR/VR) modalities allow participants to explore and experience realistic scenarios, making them useful psycho-educational tools for mental illnesses. This scoping review aims to evaluate the effectiveness of AR/VR interventions in improving (1) knowledge, (2) attitudes, (3) empathy and (4) stigma regarding people with mental illnesses. Literature on published studies in English up till April 2022 was searched within several databases. Sixteen articles were included. The majority of studies were conducted in the West (93.8%), within undergraduates (68.8%) but also amongst high school students, patients, caregivers, public including online community, and covered conditions including psychotic illnesses, dementia, anxiety and depression. A preponderance of these included studies which employed AR/VR based interventions observed improvements in knowledge (66.7%), attitudes (62.5%), empathy (100%) and reduction of stigma (71.4%) pertaining to people with mental illnesses. In the context of relatively limited studies, extant AR/VR based interventions could potentially improve knowledge, attitudes, empathy and decrease stigma regarding people with mental illness. Further research needs to be conducted in larger and more diverse samples to investigate the relatively beneficial effects of different AR/VR modalities and the durability of observed improvements of relevant outcomes of interests over time for different mental conditions.

## 1. Introduction

About 11% of the worldwide population suffer from a mental illness [1] and these mental illnesses remain the leading cause of substantial illness burden internationally in terms of disability adjusted life years [2]. Of note, people with mental illness often face challenges such as discrimination [3] and being literate in such conditions will allow people to have better understanding of people who suffer from mental illnesses [4].

Mental health literacy is defined as the knowledge and awareness of mental illness, including prevention, identification and management of these conditions [5]. Having a good level of mental health literacy can enhance the insight into mental illness, promote early help seeking, recovery and psychosocial functioning [6] and foster better attitudes towards patients with mental illness [7]. In addition, better awareness of mental illness has been associated with better employment [8], treatment adherence [9], stronger therapeutic alliance and lower clinical severity [10]. Regarding empathy, it is the ability to stand in the shoes of others and understand another’s experiences [11]. Empathy is said to have destigmatizing effects and therefore enhance positive attitudes towards people experiencing mental illness [12]. Concerning stigma about mental illnesses, it can be viewed as a set of unwarranted and negative beliefs and attitudes about mental illness, which can potentially influence discrimination, exclusion and fear of people experiencing mental illnesses [13]. 

Augmented reality (AR) uses technology to combine real and digital information so that participants experience the virtual and real contexts as one [14] and AR was used in the prominent Pokémon GO game [15]. Conversely, virtual reality (VR) excludes stimulus from the real-world setting. Virtual reality consists of two types: (1) desktop virtual reality and (2) immersive reality [16]. The former allows participants to control the virtual surroundings on a computer screen while immersive reality requires the use of a headset, earphones and controllers, which detects body movements to fully immerse participants in the virtual world. 

AR and VR (AR/VR) technologies are gaining popularity in the field of healthcare and health professions education [17] as they allow participants to immerse in realistic simulations thus serving as a useful tool in training and learning [18]. VR has also been utilized in psychotherapy for the past two decades [19]. Since then, the use of VR as a treatment modality has grown. Of note, there are recent studies examining the effectiveness of AR/VR-based therapeutic modalities in the management of patients with neurodevelopmental spectrum conditions (such as autism spectrum disorders, attention deficit hyperactivity disorders) [20,21,22,23], anxiety disorders (such as phobias) [24,25,26,27], obsessive compulsive disorder [28,29,30], post-traumatic stress disorder [31], and cognitive impairments in the elderly [32,33]. In comparison, there are fewer studies specifically elucidating the effectiveness of AR/VR based interventions as a psychoeducational tool such as in improving understanding of mental illnesses, engendering more positive attitudes regarding people with mental illnesses [34,35] and reduction of stigma [36,37].

In light of increasing interest in the use of AR/VR-based modalities within the mental healthcare setting, this scoping review aims to evaluate the effectiveness (as evidenced by improvements in ratings) of AR/VR-based interventions in improving (1) knowledge, (2) attitudes, (3) empathy and (4) stigma pertaining to people with mental illnesses.

## 2. Methodology

A scoping review is useful for exploring the literature broadly to identify the extant evidence on a specific topic of interest [38]. This scoping review was conducted according to the methodology of the Joanna Briggs Institute for scoping reviews [38]. We adopted a modified Arksey and O’Malley methodological framework for conducting scoping reviews updated by Levac *et al. (2010)* to guide the study. The first step involves identifying the main research question addressed by this review: what is the effectiveness (evidenced by improvements in ratings) of AR/VR based interventions on (1) understanding, (2) attitudes, (3) empathy and (4) stigma related to people with mental illness?

The second step involves identification of relevant studies [39]. We searched several databases (CINAHL, Cochrane Central Register of Controlled Trials, Embase, PsycINFO, Pubmed, Science Direct and Scopus) for relevant studies that examined the research question from database inception until April 2022. Keywords and combination of keywords for the literature search included: ‘virtual reality’ OR ‘(augment* reality)’ AND ‘knowledge’, ‘attitude’, ‘empathy’, ‘stigma’; ‘(virtual reality)’ OR ‘(augment* reality)’ AND ‘knowledge’ OR ‘attitude’ OR ‘empathy’ OR ‘(social distance)’ OR ‘stigma OR depression OR schizophrenia OR bipolar disorder. The inclusion criteria are as follows: (A) primary research studies on the effectiveness of AR/VR-based interventions with relevant outcomes of interest regarding people with mental illnesses, and (B) articles must be published in English. Studies were not limited by population. Studies were excluded if they were non empirical papers, opinion articles, dissertations or did not include primary outcomes of interest.

The third step involves study selection. We manually screened the abstracts of identified reports to evaluate whether they met the inclusion criteria, before reviewing full reports of these studies. Two independent reviewers simultaneously screened the titles and abstracts. In case of any inconsistency between reviewers, the disagreement was resolved by thorough discussion within the team and a third reviewer.

**Figure 1:**
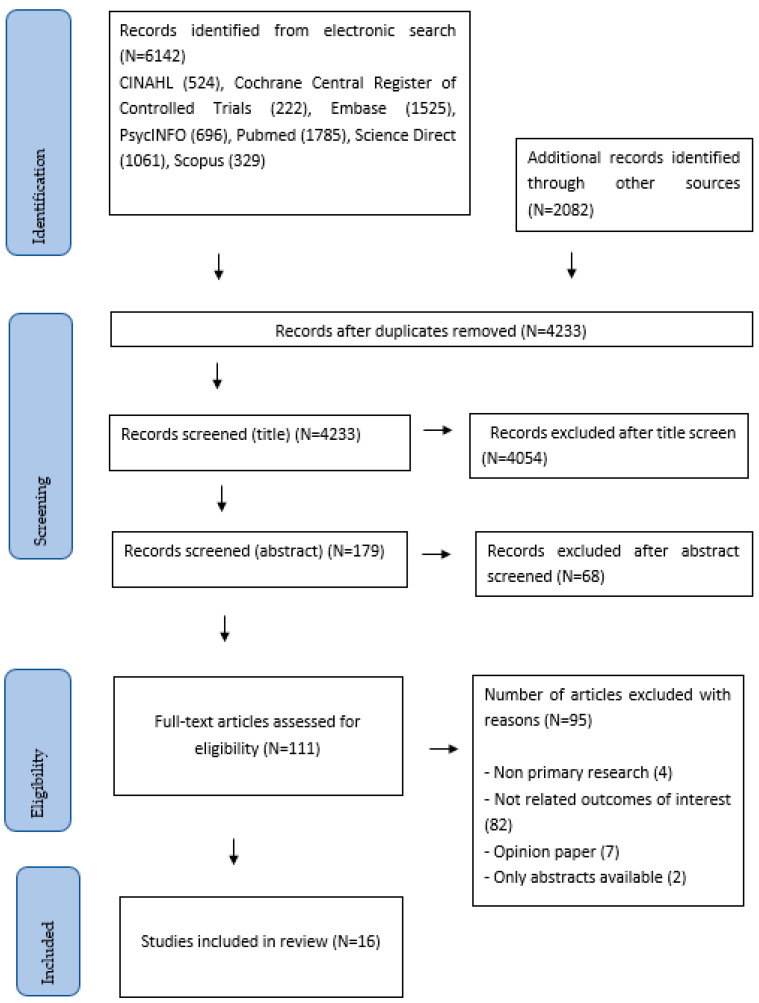
Search strategy.

The fourth and fifth steps involve charting, collating and reporting the results. For each included study, we extracted variables including the author, year of publication, characteristics of participants, AR/VR based interventions, and the main findings. The preceding data were organized and summarized into a table to facilitate independent, critical assessment by the readers. The Preferred Reporting Items for Systematic Reviews and Meta-Analyses (PRISMA) flow chart for this review is shown in Figure 1 [40]. 

## 3. Results

### 3.1. Description of Studies

Overall, 16 studies were included in this review (Table 1). The number of participants ranged from 16 to 579 in each group and included undergraduates (11 of 16 studies, 68.8%), high school students, patients, caregivers and the public, including online community. Four studies were randomized controlled trials [36,41,42,43]. Ten studies adopted quasi-experimental design. One study was a descriptive study [44] and another a prospective cohort study [45]. Six studies were conducted in United States, three studies in Australia, two studies in Spain and one study each in Brazil, The Netherlands, Germany, Ireland and Hong Kong, respectively. Only one study examined AR-based intervention [46] while the remaining 15 studies examined the use of VR, with two utilizing a Virtual Dementia Tour intervention [47,48]. AR/VR interventions ranged from virtual interactions with characters (4 of 16 studies, 25%) [36,44,49,50], environments (4 of 16 studies, 25%) [34,41,51,52] and assuming a specific character within the study (2 of 16 studies, 13%) [43,53]. Some interventions (4 of 16 studies, 25%) also allowed the participants to experience perceptual or sensory disturbances such as auditory hallucinations [35,46,47,48]. The other interventions (2 of 16 studies, 13%) allowed participants to view scenarios of characters suffering from mental illnesses [42,45]. Please see Appendix A for Cochrane’s risk of bias rating for each study.

### 3.2. Knowledge about Mental Illnesses

Nine studies examined the effects of VR interventions on knowledge and awareness of mental illness with the majority (six of nine studies) showing increased knowledge about these conditions [34,44,45,47,48,51]. In terms of nature of mental conditions, three studies examined knowledge about a range of mental illnesses [36,45,49]. Five studies examined knowledge about specific disorders, namely, psychotic conditions [34,52], dementia [47,48] and one study evaluated knowledge about both depression and schizophrenia [44]. Another study examined the effects of VR intervention on medication adherence amongst patients with schizophrenia [51]. 

In terms of nature of intervention, the study by Formosa et al. (2018) allowed participants to interact within a virtual reality intervention that simulated danger, and found significant improvement in knowledge about the psychotic disorder. Stigma-Stop is a video game that allows players to interact virtually with characters with mental illnesses. After utilizing Stigma-Stop, more than 85% of the high school students in Spain could identify panic disorder, depression and schizophrenia, although only slightly more than half could identify bipolar disorder [36]. This was largely congruent with the findings amongst psychology undergraduates in Spain [49]. Second Life (SL) simulation, involving players in a virtual reality environment, as a teaching modality, was deemed moderately effective as a psycho-educational modality [44]. In an earlier study, participants indicated greater understanding of schizophrenia, auditory and visual hallucinations after using Second Life intervention [52]. Among nursing undergraduates in the United States, the intervention group assigned with the VR case study was less likely to rate ‘do not know’ when asked about the effectiveness of hospital admission and electroconvulsive therapy indicating better knowledge [45]. One study found that reminder notes and clock in the virtual environment aided in better understanding of medication adherence [51]. Two studies used Virtual Dementia Tours whereby participants experienced changes in sensory perception while engaging in everyday tasks [47,48] with conflicting findings of improved knowledge about dementia in one study [48], but not the other [47].

### 3.3. Attitudes toward People with Mental Illnesses

Eight studies examined the effects of AR/VR based intervention on attitudes towards people with mental illness and more than half (five of eight studies) showed improvement of attitudes following the intervention [34,35,41,46,47]. In terms of the nature of mental illness, three studies examined attitudes towards a range of mental illnesses [36,45,49], three related to psychotic disorders [34,41,46] and two studies examined attitudes towards people with dementia [35,47]. 

For qualitative findings that were conducted in two studies in Spain, at least half of the participants felt that they were able to help the character with schizophrenia or bipolar and more than 80% of them felt being able to help the character with depression or panic disorder after following Stigma-Stop intervention [36,49]. In a separate study by Liu et al. (2020), participants from both VR and control groups acknowledged the need for external help beyond self, thus suggesting no difference in attitudes between the two groups.

### 3.4. Empathy

Seven studies examined empathy towards people either with dementia [47,48,50,53] or psychotic conditions [34,41,46], and found improvements of empathy across all studies following the intervention. Specific empathy scales included the Clinical Empathy Scale [34], Comprehensive State Empathy Scale [50], and 12 item Empathy Scale [41]. In the study by Wijma et al. (2018), improvement in empathy was observed in the “Perspective-taking” subscale of Interpersonal Reactivity Index but not in the “Person centeredness” subscale. 

### 3.5. Stigma Regarding People with Mental Illnesses

Seven studies examined stigma towards people with dementia [50], psychotic illnesses [41,42,46], mixed anxiety and depression [43] or a range of mental illnesses [36,49]. Most studies (five out of seven studies) found reduction of stigma for both within [36,50] and between group comparisons [36,41,43] while two studies did not [42,46].

Amongst medical students in Brazil, stigma levels were increased post-intervention [46], and students considered the VR characters with schizophrenia more dangerous than pre-intervention. Similarly, in Germany, VR intervention increased stigma when compared with both video and no intervention control groups [42].

## 4. Discussion

Overall, the majority of studies included in this scoping review were conducted in the West (93.8%), within undergraduates (68.8%) but also amongst high school students, patients, caregivers, public including online community, and covered conditions including psychotic illnesses, dementia, anxiety and depression. Amid the variety of AR/VR modalities employed within the included studies, the preponderance of these studies observed improvements in knowledge (66.7%), attitudes (62.5%), empathy (100%) and reduced stigma (71.4%) regarding people with mental illnesses. 

### 4.1. Knowledge about Mental Illnesses

We found that the AR/VR interventions used in this review were beneficial in increasing knowledge of mental illness. The VR interventions employed in the included studies allowed participants to interact with virtual characters with mental illnesses or experience perceptual or sensory abnormalities that patients with schizophrenia or dementia experience. Such patient experiences made possible through AR/VR modalities allow the participants to gain insights into the relevant mental illnesses and could impact positively on learning outcomes [54]. For example, AR/VR modalities can enhance intrinsic motivation, engagement and facilitate deeper learning in the context of life such as scenarios [54,55,56]. These benefits have rendered learning using AR/VR more fruitful than traditional learning [57]. Of note, VR have been increasingly utilized in health professions education amongst medical students, nursing students, allied health and even patients and their caregivers [58,59,60,61]. Amongst healthcare students, virtual reality interventions have been observed to improve skill competencies, and better appreciation of symptom and illness [62]. Earlier studies have found that AR/VR interventions can be adopted to manage conditions such as specific phobias, autism spectrum disorder, psychotic conditions, substance related disorders, depression and eating disorders [23,24,63,64]. In our review, we observed that VR intervention could help to assess and enhance medication adherence amongst patients with schizophrenia (Baker et al., 2006).

### 4.2. Attitude towards People with Mental Illnesses

It was observed that the AR/VR interventions improved participants’ attitudes towards people with mental illness. AR/VR-based simulations have the ability to expose participants to realistic experiences such as visual and auditory hallucinations, enhance understanding of the unique journeys that people with mental illnesses undergo and thereby foster better patient engagement [65,66]. The current findings are in agreement with that of an earlier review of AR/VR-based interventions in dementia which found that such modalities can potentially enhance knowledge and attitudes of healthcare professionals and trainees towards people with neurocognitive disorders [67]. 

### 4.3. Empathy 

It was found that the AR/VR-based interventions were largely effective in improving participants’ empathy towards people with mental illnesses. This was consistent across all intervention types including viewing of immersive VR videos of characters with mental disorders and VR interventions that allowed participants to experience perceptual disturbances. This was also consistent with earlier findings that suggested that improvements in empathy were not dependent on the nature of VR intervention [68]. The affinity between the VR character and participant was more important than the specific VR design [42,69]. Likewise, the effectiveness of the VR intervention in enhancing empathy was not dependent on whether or not the VR intervention allowed participants to adopt the protagonist character as both types of VR interventions were equally effective in some studies [34,35,44,47,51,70]. 

Empathy allows the observer to stand in the place of the other person, and experience compassion and loving kindness towards others whilst preserving one’s sense of identity, thoughts and emotions [71]. Training programs that have included the use of VR interventions have found increased empathy towards patients with specific conditions such as dementia [72], Alzheimer’s disease [73], hearing and vision loss [73] among carers, medical and nursing students.

### 4.4. Stigma

The misconception that people with mental disorders are unpredictable, violent or aggressive has been seen across societies [74]. Within this review, various VR modalities proved effective in reducing stigma towards mental illnesses, for example, Stigma-Stop (for range of mental conditions), Visit with Viv (for dementia), simulated hallucinations using VR that allowed participants to experience psychopathology (for psychotic disorders) and immersive animation in which participants played a character with mixed depression and anxiety (for affective and anxiety conditions). 

However, two interventions found an increase in stigma levels involving an AR modality with simulated hallucinations [46], and a VR intervention whereby a character spoke about his experiences with schizophrenia [42]. There are several possible reasons to explain the increase in stigma. First, mere simulation of schizophrenia may not reduce stigma unless participants can internalize such experiences and reflect on what people with mental disorders are truly experiencing [41]. Second, the construct of stigma is complex and influenced by various factors such as internalized perspectives (micro level), interactions with people with mental illnesses (meso level) and the integrated experience involving the milieu of the healthcare setting (macro level) [75]. Furthermore, these factors may also interact with other aspects such as knowledge, attitudes and aspects of empathy to affect stigma [12,76,77]. Third, the affinity between the VR character and the participants and the likeability of the VR character are likely to influence research findings [42]. Fourth, there may be participants who were uncomfortable with VR, which can overwhelm their senses. Such sensations can be intimidating, unpleasant, thus limiting the participant’s ability to engage, reflecting an increase in stigma levels [42].

## 5. Limitations and Future Research Directions

This review had several limitations. First, there were limited studies which were mostly conducted in the West amongst undergraduates, hence more future studies are warranted especially in more diverse groups internationally including within Asia. Second, the majority of the included studies had small to modest sample sizes with variable measures of outcomes. Some studies had no control group, randomization and blinding. Third, this review did not limit papers according to population, which might have contributed to the heterogeneity of the findings of included studies. Fourth, the interventions varied in terms of the types of headsets and nature of AR/VR modalities. Future research can focus on the evaluation of AR/VR-based tools in larger samples of participants (such as residents in training and healthcare professionals across different disciplines) and comparison of different AR versus VR modalities. This can be performed across different sites and adopt a standard set of rating scales over time to better evaluate the impact of such AR/VR interventions in enhancing mental health literacy, positive attitudes, empathy and reduction in stigma towards people with mental illnesses. 

## 6. Conclusions

In the context of limited studies, AR/VR based interventions were found to potentially improve knowledge, attitudes, empathy and reduce stigma related to people with mental illnesses. Further research is needed to investigate the relative beneficial effects of the different AR/VR modalities and the durability of observed improvements in outcomes of interests over time for different mental conditions.

## Figures and Tables

**Table 1:** Details of included studies.

Author Country/Setting	Design/ Participant	Intervention	Control	Outcome Measures	Knowledge	Attitudes	Empathy	Stigma
[51] United States	2 arm quasi-experimental Patients with schizophrenia and healthy control	G1: Patients with schizophrenia, *n* = 25 - Participants interact with surroundings, objects in a virtual environment using mouse. They were asked to take pills with virtual distractions (siren, ringing doorbells).	G2: Healthy control, *n* = 16 - same intervention	Pre - The Medication Management Ability Assessment (MMAA) - Continuous Performance Test	↑ Better pill selection, less time discrepancy when taking medications and greater MMAA score with reminder notes, clock	Nil	Nil	Nil
[47] United States	Pre, post single group quasi-experimental Nursing under-graduates from 3 classes	G1: Virtual Dementia Tour, *n* = 163 - equipment that altered participants’ vision, hearing and touch - complete 5 tasks - 10 min	Pre group	Pre, post - Dementia Attitudes Scale - Knowledge about Memory Loss and Care - Healthcare tour survey Post only - Students’ reflection	↑ Improvement noted post-test although it did not reach significant levels	↑ Significant improvement at post-intervention	↑ Students’ reflection highlighted that patients with dementia required empathy and sensitivity. Students affirmed the need to support caregivers and families.	Nil
[49] Spain	Post only single group quasi- experimental Psychology under-graduates aged 18 to 28	G1: Stigma-Stop, *n* = 26	Nil	Post only - Open ended questions and the following questions rated ‘yes’ and ‘no’: (1) Whether character is emotionally well? (2) Whether participant can help the character? (3) Whether participant had similar experience?	-- Most participants rated characters as emotionally unwell: a. 96%- panic disorder with agoraphobia b. 96% for schizophrenia c. 73% for bipolar disorder d. 100% for depression	-- Most participants felt they could help the character: a. 100%- panic disorder with agoraphobia b. 88% for schizophrenia c. 79% for bipolar disorder d. 92% for depression	Nil	↓ Participants’ open-ended answers showed reduction of stigma.
[36] Spain	2 arm pre post RCT High school students aged 14–18, from 24 classes	G1: Stigma-Stop, *n* = 484 from 21 classes - Non immersive virtual reality game presenting four characters experiencing depression, bipolar disorder, schizophrenia, panic disorder with agoraphobia.	G2: *n* = 68 from 3 classes	Pre, post - Student Attitudes toward Schizophrenia measures stereotypes and other aggressiveness	-- Most participants rated characters as emotionally unwell: a. 96.8%- panic disorder with agoraphobia b. 86.8% for schizophrenia c. 61.8% for bipolar disorder d. 96.1% for depression	-- About half to most of the participants felt they could help the character: a. 82.4%- panic disorder with agoraphobia b. 62.5% for schizophrenia c. 53% for bipolar disorder d. 90% for depression	Nil	↓ Stigma-Stop group had significantly lower stigma
[34] Australia	Pre-post single group quasi- experimental Public and psychology under-graduates	G1: VR, *n* = 50 - with head mounted display, headphones and Xbox controller, - virtual environment with VH environmental sounds and voices suggestive of danger - 10–15 min	Pre group	Pre, post - Knowledge * about psychosis - Attitude ^ by Reavley and Jorm (2011) - Empathy by clinical empathy scale	↑ Significant improvement at post-test	↑ Significant improvement in attitude scores at post-test	↑ Significant improvement in empathy scores at post-test	Nil
[35] Australia	Pre-post 2 arm quasi-experimental Medicine and pharmacy under-graduates	G1: Australia Vic Virtual Dementia Experience, *n*= 80 - multisensory, virtual simulation to allow participants to experience perceptual and cognitive difficulties by patients - 1.5 h	G2: Waitlist control group, *n* = 198 - curriculum as usual	Pre post - Dementia Attitudes Scale	Nil	↑ Intervention group had significantly better attitude scores at post-test	Nil	Nil
[44] United States	Descriptive study Nursing students, *n* = 126	G1: VR condition, *n* = 126 - Students viewed virtual neighbourhood with two houses: one belonged to someone with schizophrenia, another with depression. Students enter house and interact with patient. - Students were given case studies on the patients. - 45–60 min	Nil	Post only - * 35-item Second Life (SL) Simulation Evaluation Survey - Two open-ended questions regarding feedback about stimulation	↑ Second Life Simulation, as a teaching modality, was moderately effective.	Nil	Nil	Nil
[45] United States	Pre- and post-intervention study 4th year nursing undergraduates	G1: VR, *n* = 149 - one virtual simulation case study weekly on depression, bipolar disorder, anxiety, alcohol withdrawal and schizophrenia - 30 min, student can repeat simulation	G2: Non-simulation group, *n* = 150	Post only - Two vignettes, schizophrenia and depression. Participants answer about their perception of helpfulness of certain people (including healthcare workers, traditional healers, family and friends), specific medications and interventions (e.g., physical activity, massage, relaxation, specific therapies).	↑ For perceived helpfulness of pharmacological interventions, intervention group was less likely to rate antipsychotics and sleeping pills as ‘do not know’ for the depression vignette. Control group was more likely than G1 to rate psychiatric hospital admission and electroconvulsive therapy as ‘do not know’.	-- Both groups agreed solving the problem by self as unhelpful.	Nil	Nil
[50] Australia	Pre post single group quasi-experimental Students and public	G1: Visit with Viv, *n* = 35: - VR about Viv, who recounts her experiences of dementia > life-size in art gallery, > Occulus Quest VR headset in university, *n* = 36 - 15–20 min	Pre group	Pre post - comprehensive state empathy scale - Change in emotional distance scale	Nil	Nil	↑ Significant improvement in empathy scores from pre-test	↓ Significant reduction in stigma levels at post-test
[46] Brazil	Pre post single group quasi-experimental Medical students from 3 universities	G1: AR, *n* = 21 - figures and voices from narratives of three patients with schizophrenia - voices included whispers, commanding and threatening speech - 3 min	Pre group	Pre post - Schizophrenia stigma * Post only - Evaluate environment simulation	Nil	↑ Significant increase in the average score of help-giving at post-test	↑ Increase in empathy	↑ Increase in stigma especially in fear, pity and segregation.
[48] Ireland	Pre post repeated single group quasi-experimental Health professionals, voluntary groups and public	G1: Virtual Dementia Tour + watching another group doing distortion session, *n* = 240 - 2 h - 10 min of sensory distortion - 30 min debriefing	Pre group	Pre post, follow up at 3 months * Tool that measures empathy, understanding of behaviours and role of the person in care decisions.	↑ Significant improvement in understanding of behavioural impact of dementia across time points	Nil	↑ Significant improvement in empathy across time	Nil
[41] United States	4 arm post only RCT Psychology research participant pool and university community	G1: VR condition, *n* = 26 - Participant plays the character of someone experiencing schizophrenia, visiting a pharmacist asking for prescription refill. - 4.5 min G2: Empathy condition, *n* = 26 - Participants were asked to pen their thoughts about experiencing VH and AH while getting prescription - 1 min G3: VR+ empathy condition, *n* = 26 - Empathy before VR condition	G4: control, *n* = 26 No intervention control group	Post only - Empathy 12-items - Social Distance Scale - Attitudes Toward People with Schizophrenia, 7 items - Evaluation of simulation - Pre-existing attitudes towards people with schizophrenia, 8 items	Nil	↑ Intervention group had better attitudes but this did not reach significant levels	↑ Significant improvement in empathy in intervention groups using VR	↓ VR group had significantly lower stigma
[42] Germany	3 arm post only RCT Majority are students from university, *n* = 114	G1: VR, *n* = 31 - Young male actor speaking about his experiences with schizophrenia, including how his loved ones cope with it. G2: regular video, *n* = 45 - Similar as the VR but fixed perspective	G3: No intervention control group, *n* = 38	Post only - Stigma with four related constructs: anxiety *, social proximity ^, empathy (by Kinnebrock et al., 2010, - Benevolence (using Community-Attitudes-Toward-the-Mentally Ill Inventory)	Nil	Nil	Nil	↑ VR contact did not decrease stigmatization compared with control but had increased stigmatization compared with video.
[53] The Netherlands Alzheimer’s Society	Pre post single group quasi-experimental Informal caregivers caring for those with dementia	G1: VR, *n* = 35 360-degree simulation movie on virtual reality (first person view) and e-course, *n* = 42- different scenes of interactions e.g., confronted by daughter about remote control in cupboard and she talks to people on the phone about you - 13 min	Pre group	Pre, post - Empathy measured by Person-centeredness subscale of Approach to Dementia questionnaire and ‘perspective-taking’ subscale of Interpersonal Reactivity Index	Nil	Nil	↑ Significant improvement using the perspective-taking subscale	Nil
[52] United States	Post only single group quasi- experimental Second Life Users	G1: Second Life VR, *n* = 579 - Character toured environment, experiencing hallucinations including voices, posters changing text to profanities, floor that fall away, TV that encourage suicide and gun with voices telling character to commit suicide, and own reflection with bleeding eyes.	Nil	Post only - Questions about understanding of hallucinations	-- Intervention group had improvements of understanding of (1) AH- 76.86% (2) VH- 69.91% (3) Schizophrenia- 73.9%	Nil	Nil	Nil
[43] Hong Kong	3 arm pre post RCT University students 18 years old and above	G1: Immersive animation, *n* = 82 Participants played a character, Yan, who had mixed anxiety and depression, and who was speaking with an uncle. Pop up messages illustrated problems in Uncle’s communication. G2: Text condition, *n* = 80 Participants read same story in 2D effect, without immersive experience. All 3 groups: 10 min, had VR headset, Oculus Go.	G3: Control, *n* = 82 Exoplanet VR video, 360°	Pre, post, 1 week follow up: - Stigma by 21-item Public Stigma and Acceptance Scale - 7-item Sense of Embodiment Scale * - Story Transportation	Nil	Nil	Nil	↓ Immersive animation and text condition had significantly lower public stigma at post-test and follow up compared with the control group. Immersive animation vs. text condition did not have significantly different stigma levels between them.

AH = auditory hallucinations; AR = augmented reality; diff = difference; G = group; M = Mean; MMAA = Medication Management Ability Assessment’ min = minutes; sig = significantly; VH = visual hallucinations; * constructed by authors; ^ modified by authors, ↑ = increased, ↓ = decreased, -- = non-quantitative measurement.

**Figure A1:**
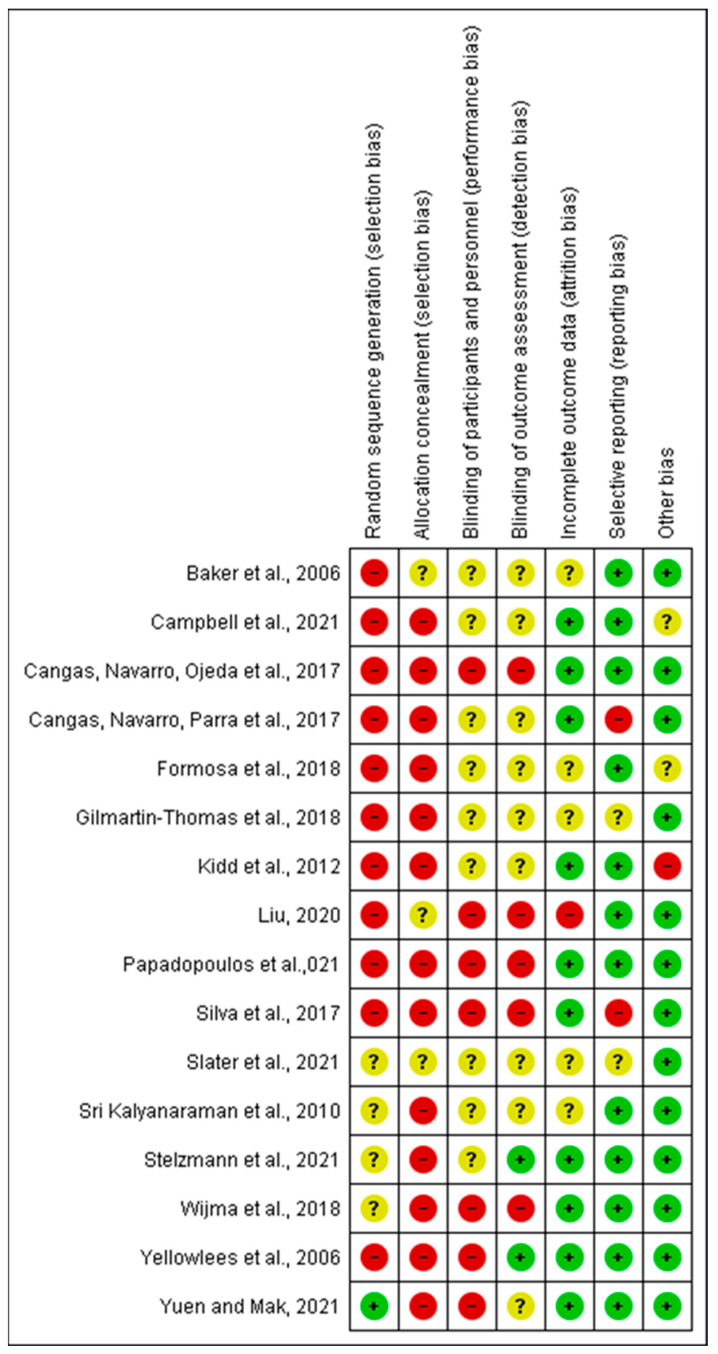
(+): low risk of bias, (-): high risk of bias, (?): unknown risk of bias [34,35,36,41,42,43,44,45,46,47,48,49,50,51,52,53].

## Data Availability

The data presented in this study are available on request from the corresponding author.
